# Tumor organoid-immune co-culture models: exploring a new perspective of tumor immunity

**DOI:** 10.1038/s41420-025-02407-x

**Published:** 2025-04-24

**Authors:** Jing Wang, Xiaoyue Tao, Jialong Zhu, Zhe Dai, Yuanyang Du, Yiyang Xie, Xiaoyuan Chu, Gongbo Fu, Zengjie Lei

**Affiliations:** 1 Department of Oncology, Jinling Clinical Medical College, Nanjing Medical University, Nanjing, China; 2 Department of Oncology, Jinling Clinical Medical College, Nanjing University of Chinese Medicine, Nanjing, China; 3 Department of Oncology, Nanjing Jinling Hospital, Affiliated Hospital of Medical School, Nanjing University, Nanjing, China; 4 Department of Oncology, Jinling Hospital, The First School of Clinical Medicine, Southern Medical University, Nanjing, China

**Keywords:** Experimental models of disease, Cancer models

## Abstract

Recent advancements in technology have significantly expanded the scope of tumor research, progressing from the study of individual cells to more intricate tissue and organ-level analyses. Tumor organoids have emerged as a highly realistic platform for investigating tumor growth, development, and their interactions with the surrounding microenvironment. However, a notable limitation of these organoids is their lack of the diverse cellular composition typically observed in actual tumors, which hinders their ability to fully replicate the complexity of the tumor microenvironment. Immune cells play a pivotal role, and tumor immunology has become a major research hotspot. Research in tumor immunology aims to elucidate how the immune system recognizes and attacks tumor cells, as well as how tumor cells evade immune surveillance. In recent years, there has been growing interest in co-culturing immune cells with tumor organoids, an approach that has yielded valuable insights into the intricate interactions between tumors and the immune system. The aim of this paper is to review and discuss the progress achieved in co-culturing tumor organoids with immune cells. By doing so, we hope to offer a new perspective and enhance our understanding of the complexity and diversity inherent in the tumor microenvironment.

## Facts

- The article provides an overview of recent advancements in co-culturing tumor organoids with immune cells.
- These co-culture models offer valuable insights into the complex interactions between tumors and the immune system.
- Co-culture models serve as a more physiologically relevant in vitro model.

## Open questions

- How can we improve the success rate of co-culturing tumor organoids with immune cells?
- What critical roles do tumor organoid-immune cell co-culture models play in advancing cancer diagnosis and treatment?
- What are the key challenges and strategies for effectively translating tumor organoid-immune cell co-culture models into clinical applications?

## Introduction

Tumors represent a significant and widespread challenge to human health, characterized by the coexistence of mutated tumor cells and non-tumor cells within the tumor tissue. These non-tumor cells include immune cells, vascular cells, and stromal cells, which together with tumor cells form the complex and dynamic tumor microenvironment [1]. The tumor microenvironment plays a pivotal role in tumor initiation, progression, and response to therapeutic interventions. As such, a comprehensive understanding of tumors requires an in-depth exploration of the intricate interactions and mechanisms within the tumor microenvironment. Organoids are three-dimensional cellular models that are cultured in vitro and replicate critical features of human organs, including their structural, genetic, and functional characteristics. They offer an excellent foundation for stem cell research, regenerative biology, organogenesis, precision medicine, and the study of human pathology [2]. Tumor organoids, in particular, are derived from cancer cells isolated from patient tumor tissues. They closely mimic the biological properties and drug responses of the primary tumors, a valuable tool in cancer research and personalized medicine [3]. By studying tumor organoids, we can gain a better understanding of the mechanisms behind tumor development and explore more effective treatment strategies. However, the lack of a tumor microenvironment in these models has been a limiting factor. To overcome this challenge, researchers have developed advanced co-culture models that incorporate various cell types to better simulate the tumor microenvironment. These enhanced models provide a more physiologically relevant and comprehensive platform for studying the diverse characteristics and behaviors of different types of cancer.

Tumor immunity is a complex process in which immune cells play a pivotal role within the tumor microenvironment, engaging in multifaceted interactions with tumor cells [4]. Co-culturing tumor organoids with immune cells has emerged as an innovative research strategy to model the dynamic interplay between tumors and the immune system. This approach enables researchers to observe how immune cells influence tumor growth and progression. For instance, Dijkstra et al. developed a co-culture platform combining peripheral blood lymphocytes and tumor organoids. This platform was utilized to enrich tumor-reactive T cells from the peripheral blood of patients with mismatch repair-deficient colorectal cancer (CRC) and non-small cell lung cancer. Their findings demonstrated that these T cells could effectively assess the cytotoxic efficacy against matched tumor organoids. Moreover, their work established a methodology to evaluate the sensitivity of tumor cells to T cell-mediated attacks at an individualized patient level [5]. Tsai et al. constructed a co-culture model involving peripheral blood mononuclear cells and pancreatic cancer organoids. Through this model, they observed the activation of myofibroblast-like cancer-associated fibroblasts and tumor-dependent lymphocyte infiltration [6]. These studies offer valuable insights into the complex interactions between tumors and the immune system, providing a deeper understanding of the mechanisms underlying tumor immunity [7].

The co-culture of tumor organoids and immune cells not only has a profound impact on understanding tumor biology but also plays a key role in promoting the innovation of cancer treatment. By simulating the tumor microenvironment within the body, this approach enables the exploration of intricate interactions between tumors and the immune system, thus providing a theoretical basis for the development of more effective immunotherapy. In this review, we provide an overview of the components and current state of tumor organoid-immune co-culture models that have emerged in recent years. We also examine their applications and the functional assays employed in these co-culture systems, while addressing the challenges and limitations associated with their implementation and future use. We anticipate that the continued advancement and application of tumor organoid-immune co-culture models will significantly enhance their impact on both biological discoveries and clinical translation.

## Tumor organoids

In recent years, significant progress has been made in the field of tumor research, particularly in the study of tumor organoids [8]. Tumor organoids have emerged as a research hotspot, serving as three-dimensional cell models cultured in vitro that better mimic the biological characteristics of tumors in vivo. These organoids are constructed from tumor cells or cells derived from patients and exhibit morphological, genetic, and functional features similar to those of primary tumors, allowing for the accurate representation of tumor heterogeneity. Tumor organoids provide a novel platform for investigating the pathogenesis of tumors, drug screening, and prediction of treatment responses [9, 10]. The establishment of tumor organoids primarily depends on advanced cell culture techniques and the use of a supportive extracellular matrix (ECM). Researchers commonly use biocompatible ECMs, such as Matrigel, as the culture substrate for these organoids. This type of matrix can provide the necessary growth and survival signals to the cells while preserving their three-dimensional structure and functionality [11]. Furthermore, researchers are continually refining culture conditions and optimizing medium formulations to enhance the growth and stability of tumor organoids.

Compared to primary tumor cells, tumor organoids encompass the source tumor cells along with their corresponding genetic characteristics. The establishment of different tumor organoid models requires distinct culturing methods, and currently, there are no internationally standardized culture media or techniques. Typically, the optimal tissue source for obtaining tumor cells is from the tumor margin, with minimal necrosis rates [12]. The establishment process of tumor organoids generally begins with the mechanical dissociation and enzymatic digestion of tumor samples, followed by seeding the cell suspension onto biomimetic scaffolds such as Matrigel. Matrigel primarily consists of adhesive proteins, proteoglycans, and collagen IV, which provide structural support for the cellular architecture of organoids. The cultivation of tumor organoids often employs growth factor-reduced media to minimize clone selection and avoid potential confounding effects on drug treatments. Commonly used growth factors in the culture medium for maintaining tumor organoids include Wnt3A, R-spondin-1, TGF-β receptor inhibitors, epidermal growth factor, and Noggin. The specific combination and concentration of these factors depend on the tumor type being cultured [12, 13]. Various culturing methods for tumor organoids have been developed, covering a wide range of solid tumors such as colorectal cancer [14], breast cancer [15], hepatocellular carcinoma [16], prostate cancer [17], and non-small cell lung cancer [18], among others. Most tumor organoids are derived from patient cancer samples and are generated under conditions that support adult stem cell-based organoid growth [13]. Although less common, there are also studies that utilize the CRISPR-Cas9 gene editing system to establish tumor organoids [9, 19]. Furthermore, research efforts are exploring alternative sources for constructing tumor organoids, such as circulating tumor cells, urine, and bronchoalveolar lavage fluid, beyond traditional tissue samples [20].

The development of tumor organoids has made significant progress, establishing them as a widely accepted model in cancer research. These organoids have demonstrated broad applicability across various fields, including tumor biology, drug discovery, and individualized therapy. By utilizing tumor organoids for drug screening, researchers can evaluate the toxicity and efficacy of different drugs on tumor cells, offering valuable insights for clinical treatment. Moreover, tumor organoids serve as a powerful tool for studying tumor metastasis and mechanisms of drug resistance, laying the theoretical foundation for the development of new therapeutic strategies (Table 1). To date, diverse patient-derived tumor organoid models have been developed to evaluate the effects of different immunotherapies [3]. In addition, from 2017 to 2023, 42 clinical trials have used tumor organoids derived from cancer patients to aid in optimizing clinical decision-making [21]. Despite their widespread adoption and utility in cancer research, tumor organoids are not without limitations. Tumor organoids typically consist of a single epithelial layer and lack the tumor microenvironment components such as surrounding stroma, immune cells, blood vessels, and the nervous system [3, 20]. Therefore, the development of co-culture models of tumor organoids with other cells may be able to replicate the intercellular interactions, which has become a research hotspot.

**Table 1:** Technological overview of co-culture models.

Organoid type	Components used	Platform and matrix	Type of disease	Main outcomes	Detection technology	Reference
Cholangiocarcinoma organoid	T cell	Basement membrane extract-mediated direct co-culture	Cholangiocarcinoma	Allowing for the study of interactions between cholangiocarcinoma organoids and immune cells, as well as the evaluation of immunotherapies.	ATP quantification cell viability assay, HepG2 killing assay, Flow cytometry, Confocal time-lapse imaging and analysis, RNA sequencing analysis	[38]
Pancreatic ductal adenocarcinoma organoid	T cell	Matrigel-mediated direct co-culture	Pancreatic ductal adenocarcinoma	A resource for investigating pancreatic cancer and its microenvironment.	Flow cytometry, Immunofluorescence, Organoid histology, Immunohistochemistry, RT-PCR	[6]
Lung cancer organoid	T cell	Gel-liquid-interface co-culture model on a superhydrophobic microwell array chip	Lung cancer	Gel-liquid-interface co-culture can be used for developing diagnostic strategies for precision immunotherapies as well as understanding the underlying mechanisms.	Flow cytometry, qRT-PCR, Immunofluorescence, Drug sensitivity assays, Single-cell preparation and transcriptome library construction, RNA sequencing analysis	[47]
Clear cell renal cell carcinoma organoid	T cell	Microfluidic chip	Clear cell renal cell carcinoma	It offers insights into the rational design and optimization of viral-based immunotherapies for Clear cell renal cell carcinoma.	Oncolytic adenoviruses,Microfluidic chip assay,Immunofluorescence	[48]
Pancreatic ductal adenocarcinoma organoid	T cell	Matrigel-mediated direct co-culture	Pancreatic ductal adenocarcinoma	The combination of ultradeep single-cell sequencing and organoid techniques enabled rapid characterization of tumor-responsive T cell receptors for developing practical personalized T cell receptor-T therapy.	Immunohistochemistry, Immunofluorescence, RNA sequencing analysis	[50]
Lung adenocarcinoma organoid	T cell	Matrigel-mediated direct co-culture	Lung adenocarcinoma	The PDO model cocultured with autologous peripheral blood mononuclear cells can potentially be used to determine the optimal immune-priming strategy for individual patients with Lung adenocarcinoma.	Drug sensitivity assays, Immunofluorescence, CD8 + T cell activation and PDO-killing assays	[45]
Melanoma organ oid and breast cancer organoid	T cell	Acoustic virtual 3D scaffold	Melanoma	The Acoustic virtual 3D scaffold offers an efficient method for tumor organoid–immune cell studies, advancing cancer research and immunotherapy development.	ELISA, Flow cytometry, Immunohistochemistry, RNA sequencing analysis	[49]
			Breast cancer			
High‐grade squamous intraepithelial lesion organoid and Squamous carcinoma organoid	T cell	Basement membrane extract-mediated direct co-culture	High‐grade squamous intraepithelial lesion	An experimental platform and biobanks for in vitro mechanistic research, vaccine development, targeted therapy research, and personalized treatment of human papillomavirus‐related cervical diseases.	Immunohistochemistry, Immunofluorescence, RNA sequencing analysis, Whole-Genome DNA Analysis, Drug sensitivity assays, Lentiviral Transduction, Live Imaging of Spheroid Apoptosis	[44]
			Squamous Carcinoma of the Cervix			
Lung cancer organoid	T cell	Modular superhydrophobic microarray chip	Lung cancer	A joint phenotypic and transcriptomic function-associated single-cell RNA sequencing platform to evaluate novel immunotherapies and to understand the underlying molecular mechanisms.	Drug sensitivity assays, Flow Cytometry, RNA sequencing analysis	[46]
Colorectal cancer organoid	CD8 + T cell	Matrigel-mediated direct co-culture	Colorectal cancer	High intratumoral Fusobacterium nucleatum predicts favorable response to anti-PD-1 therapy as a potential biomarker of immunotherapy response in microsatellite stable colorectal cancer.	Multicolor flow cytometry analysis of immune cells	[40]
Pancreatic ductal adenocarcinoma organoid	CD8 + T cell	Matrigel-mediated direct co-culture	Pancreatic ductal adenocarcinoma	Targeting Myeloid-derived suppressor cells could be a potential therapeutic strategy for Pancreatic ductal adenocarcinoma treatment.	Flow Cytometry, RT-PCR, Immunofluorescence, Immunohistochemistry, NanoString Technologies Digital Spatial Profiling	[39]
Gastric cancer organoid	CD8 + T cell	Cell-basement membrane matrix-mediated direct co-culture	Gastric cancer	The co-culture model improves the outcome of cancer patients by predicting the response to immunotherapy.	Immunofluorescence, Time-lapse microscopy	[41]
Colorectal cancer organoid,normal colon or non-small cell lung cancer organoid	T cell	96-well U-bottom plate-mediated direct co-culture	Colorectal cancer	This platform provides a clinically feasible strategy for generating patient-specific T cell products for adoptive T cell transfer.	Flow cytometry, Organoid killing assays, Immunohistochemistry, Whole-exome DNA Analysis, T cell recognition assays	[5]
			Non-small-cell lung cancer			
Glioblastoma organoid	CAR-T cell	24-well ultra-low-attachment culture plate-mediated direct co-culture	Glioblastoma tumor	Through the analysis of individualized reactions and the application of genetically engineered CAR T cells for precise cancer therapies.	Immunohistochemistry, ELISA assay, Caspase 3 staining	[51]
Pancreatic ductal adenocarcinoma organoid	CAR- T cell	center gel region of the 3D microfluidic culture device	Pancreatic ductal adenocarcinoma	CAR-T cells enable modification of tumor stroma, leading to increased elimination of pancreatic ductal adenocarcinoma tumors.	Luciferase killing assay, Real-time cytotoxicity assay, Co-culture repeat stimulation assay, Confocal live cell, Time-lapse microscopy	[53]
Glioblastoma organoid	CAR- T cell	24-well ultra-low-attachment culture plate-mediated direct co-culture	Glioblastoma	The ability to target patient-derived glioblastoma underscores the translational significance of this EphA3 CAR T-cell therapy in glioblastoma treatment strategies.	Gene expression analysis, Cytotoxicity assay, Cytokine assay, Flow cytometry	[57]
Breast cancer organoid	CAR- T cell	Breast cancer-on-chip	Breast cancer	The modular architecture of the tumor-on-chip paves the way for studying the role of other cell types and thus accelera the preclinical development of CAR-T cell products.	Dasatinib treatment, Cytokine assay, Image acquisition and analysis, Flow cytometry	[54]
Glioblastoma organoid	CAR- T cell	CytoView plates direct co-culture	Glioblastoma	Findings highlight a unique trial design as a valuable platform for real-time assessment of CAR-T cell bioactivity and insights into immunotherapy efficacy.	Tumor Cytolysis Assays, Immunohistochemistry, Flow cytometry, Cytokine assay	[55]
Bladder cancer organoid	CAR-T cell	Matrigel-mediated direct co-culture	Bladder cancer	Outlining a comprehensive workflow for personalized CAR-T cell therapy testing.	Immunofluorescence, Whole-exome DNA analysis, RNA sequencing analysis, Flow cytometry, RT-PCR, Cytokine assay	[52]
Hepatocellular carcinoma organoid	CAR-T cell	Matrigel-mediated direct co-culture	Hepatocellular carcinoma	CAR-T cells demonstrated robust recognition and elimination of carcinoma cells, exhibiting enhanced persistence and cytotoxicity compared to conventional T cells.	Whole-exome DNA analysis, qPCR, Flow cytometry, Enzyme-linked immunosorbent spot, Cytotoxicity assay, Immunofluorescence	[56]
Colorectal cancer organoid	CAR-NK cell	Reduced Growth Factor Basement Membrane extract-mediated direct co-culture	Colorectal cancer	NK cells can efficiently infiltrate and kill tumor organoids in a CAR-dependent manner.	Immunoblot analysis, qRT-PCR, Flow cytometric, Cytotoxicity analysis	[14]
Colorectal cancer organoid	TIL	Matrigel-mediated direct co-culture	Colorectal cancer	Cancer cells acquire immune regulatory molecules from CD4 T cells through trogocytosis.	Flow cytometry, Confocal microscopy, RNA sequencing analysis, RT-PCR	[61]
Melanoma organoid	TIL	Air-liquid culture and matrigel-mediated direct co-culture	Melanoma	TILs significantly expanded under the co-activation of IL-2 and anti-PD-1 antibodies.The small molecule Navitoclax can enhance the immunotherapy effects of TILs.	Immunofluorescence, Flow cytometric, Apoptosis and Cytotoxicity analysis	[62]
Cervical cancer organoid	TIL	Matrigel-mediated direct co-culture	Cervical cancer	Established a patient-derived organoid cervical cancer biobank.Successfully evaluated the responses of different organoid samples to TILs.	Immunohistochemistry, Immunofluorescence, Genomic analysis, Drug sensitivity assays, Organoids killing assay, Flow cytometry	[63]
Colorectal cancer organoid	TIL	Matrigel-mediated direct co-culture	Colorectal cancer	Provided an approach to generate tumor-specifc T cell receptors from tumor-infltrating lymphocytes of patients, used for personalized T cell receptor-engineered T cell therapy.	ELISpot, Flow cytometric, RT–PCR, ELISA assay, Cytokine assay	[64]
Bladder cancer organoid	NK cell	96-well plate-mediated direct co-culture	Bladder cancer	NK cells recruit T cells by secreting a panel of chemokines for Non-muscle-invasive bladder cancer treatment.	Cell counting kit-8 assay, Degranulation assay, Cytokine assay, Flow cytometric, RNA sequencing analysis, Transwell chem otaxis assay	[65]
Mouse mammary organoid and breast cancer organoid	NK cell	Matrigel-mediated direct co-culture	Breast cancer	Using tumor organoids and ex vivo assays to uncover intrinsic cancer cell mechanisms driving the metastatic cascade.	Antibody dependent cell mediated cytotoxicity assay	[67]
Breast cancer organoid	NK cell	Matrigel-mediated direct co-culture	Breast cancer	Tumor-educated NK cells exhibit distinct gene expression profiles and functional properties compared to NK cells from healthy breast tissue.	Flow cytometric, Tail vein assay, Immunofluorescence, qPCR, RNA sequencing analysis and gene-set analysis	[68]
Pancreatic ductal adenocarcinoma organoid	NK cell	Matrigel-mediated direct co-culture	Pancreatic ductal adenocarcinoma	The concentration of serum NKG2D ligands, particularly ULBP2, is an independent marker of survival in Pancreatic ductal adenocarcinoma patients.	Immunophenotyping, Cytotoxicity assay, Cytokine assay, qRT-PCR, Immunohistochemistry	[69]
Squamous cell carcinoma organoid	Macrophage	Collagen-I gel-mediated direct co-culture	Skin squamous cell carcinoma	Exploring how macrophage polarization (M1 or M2) affects tumor progression.	Immunohistochemistry, Immunofluorescence, In Situ Zymography	[74]
Hepatocellular carcinoma organoid	Tumor-associated macrophage	Microfluidic chip	Hepatocellular carcinoma	This coculture approach enhances the prediction of patient responses to immunotherapies.	Drug sensitivity assays, Immunohistochemistry, Immunofluorescence, RNA sequencing analysis	[75]
Colorectal cancer organoid	Macrophages	24-well Transwell	Colorectal cancer	SIRT1-positive colorectal cancer promotes tumor-associated macrophage migration, influences the CXCR4/CXCL12 pathway.	Flow cytometric, qRT-PCR, Immunofluorescence, Immunohistochemistry, Western blot, RNA sequencing analysis	[76]
Pancreatic adenocarcinoma organoid	Macrophages	6/24-well Transwell	Pancreatic adenocarcinoma	Using this macrophage-organoid co-culture model, the authors elucidated the involvement of the macrophage-CCL5-Sp1-AREG loop in the interplay between macrophages and pancreatic cancer cells.	Cell viability,Flow cytometric,Sphere formation,ELISA assay,Western blot,Immunofluorescence	[77]
Pancreatic ductal adenocarcinoma organoid	Bone marrow-derived dendritic cell	The round-bottom ultra-low attachment 96-well plate direct co-culture	Pancreatic ductal adenocarcinoma	Demonstrates the impact of dynamic glycolytic reprogramming on the composition of immune cells in the tumor microenvironment of pancreatic ductal adenocarcinoma, especially on the antigen-presenting function of dendritic cells.	Air–Liquid Interface co-culture, Proliferation assay and ATP measurement, qRT-PCR, Western blot	[80]
Colorectal cancer organoid	DC	Bovine Collagen type I-mediated direct co-culture	Colorectal cancer	Suggesting potential therapeutic avenues for addressing tumor-driven DC dysfunction and enhancing anti-tumor immune responses.	Flow cytometric, Immunofluorescence, Immunohistochemistry, Flow cytometric	[81]
Colorectal cancer organoid	DC	Bovine Collagen type I- mediated direct co-culture	Colorectal cancer	The phenotypic switch of cDC2 is involved in the disruption of the immune system, impacting the efficacy of anti-tumor immunotherapy.	Immunofluorescence, Immunohistochemistry, Flow cytometric, ELISA assay, Cytokine assay	[82]
Glioblastoma organoid	DC	24-well Transwell	Glioblastoma	The mechanism of DC-CIK cells on glioblastoma cell models is characterized by a significant increase in apoptosis and elevated secretion levels of IFN-γ.	Cytotoxicity assay, Apoptosis assay, Immunohistochemistry, qRT-PCR, ELISA assay	[83]
Melanoma organoid	DC	Matrigel-mediated direct co-culture	Melanoma	Utilized co-culture model of melanoma organoids with DC to validate that a nanovaccine could effectively stimulate the maturation of bone marrow-derived DC.	Cell counting kit-8 assay, ELISA assay, Flow cytometric, Cytotoxicity assay, Immunofluorescence, Immunohistochemistry,	[84]

## Tumor immunity

The tumor immune system is a vital component of the tumor microenvironment, playing a critical role in identifying and eliminating tumor cells. Composed of diverse cells and molecules, the tumor immune system cooperates synergistically to counteract tumor development and progression [4]. At the core of the tumor immune system are immune cells, classified into two main categories: adaptive immune cells and innate immune cells. Adaptive immune cells are activated upon exposure to specific antigens and utilize immune memory to amplify immune responses [22]. Key players in the adaptive immune system include T cells and B cells. In contrast, innate immunity, also referred to as intrinsic immunity, is a non-specific defense mechanism that becomes active within hours of foreign antigen entry into the body. Cells responsible for executing innate immune responses include macrophages, natural killer (NK) cells, neutrophils, and dendritic cells [23]. In addition to immune cells, the tumor immune system also encompasses a variety of molecules and signaling pathways. One significant molecule is TNF, which can induce apoptosis in tumor cells, trigger inflammatory responses, and enhance the activity of immune cells. Another key signaling pathway is the cytokine-mediated immune response, where cytokines such as interferons and interleukins activate immune cells, boosting their capacity to attack tumor cells [24]. The tumor immune system contributes to anti-tumor immune responses in multiple ways. First, immune cells directly target and destroy abnormal cells by recognizing and eliminating tumor cells. Second, they produce cytokines and antibodies that inhibit tumor growth and metastasis [25]. Furthermore, the tumor immune system possesses memory capabilities; once immune cells identify and eliminate tumor cells, they retain information about the specific antigen, enabling a swift and effective response upon subsequent encounters.

Immune cells play a pivotal role in the tumor microenvironment. However, the tumor immune system also faces challenges and limitations. Although tumor-antagonizing immune cells often target and kill cancer cells in the early stages of tumor development, cancer cells appear to eventually escape immune surveillance and even suppress the cytotoxic effects of tumor-antagonizing immune cells through various mechanisms [26]. The ability of immune evasion serves as a novel hallmark of cancer, providing opportunities for new strategies in cancer therapy that harness the combat capabilities of immune cells against cancer cells. Recently, immune checkpoint inhibitors, particularly those targeting CTLA-4 and PD-1/PD-L1 pathways, have shown remarkable anti-tumor effects across various cancer types, marking a transformative era in cancer treatment. These inhibitors work by blocking the mechanisms tumor cells use to evade immune detection, thereby restoring the anti-tumor activity of T cells and enhancing the patient’s immune system’s ability to attack tumors effectively [27, 28]. Within the tumor microenvironment, non-immune cells, including endothelial cells, fibroblasts, and neural cells, also contribute significantly to tumor progression. Vascular endothelial growth factor receptor on vascular endothelial cells promotes angiogenesis and invasiveness, fostering a tumor-promoting effect in the tumor microenvironment [29]. In glioblastoma multiforme, studies have demonstrated that tumor cells preferentially grow and migrate along nerve fibers within the tumor microenvironment [30]. In addition, fibroblasts not only directly support tumor growth and invasion but also indirectly promote tumor development by regulating immune response and matrix remodeling [31–33].

## Co-culture of tumor organoids with immune cells

**Fig. 1:**
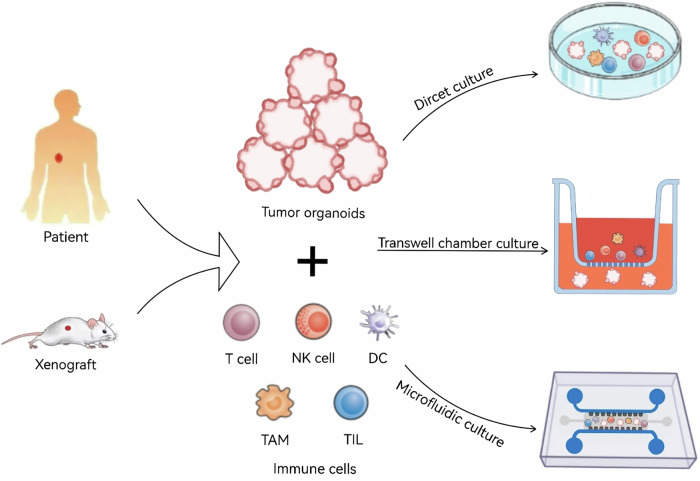
Co-culture of tumor organoids and immune cells. Tumor tissue obtained from surgical resection or living tissue is mechanically and enzymatically digested into cells, then seeded in a biocompatible extracellular matrix, and cultured in a medium supplemented with specific growth factors. Tumor organoids and immune cells are co-cultured in various ways, including direct co-culture [85], Transwell-based co-culture [87], and co-culture based on microfluidic chip technology [88], to simulate the interaction between tumor and immune cells.

**Fig. 2:**
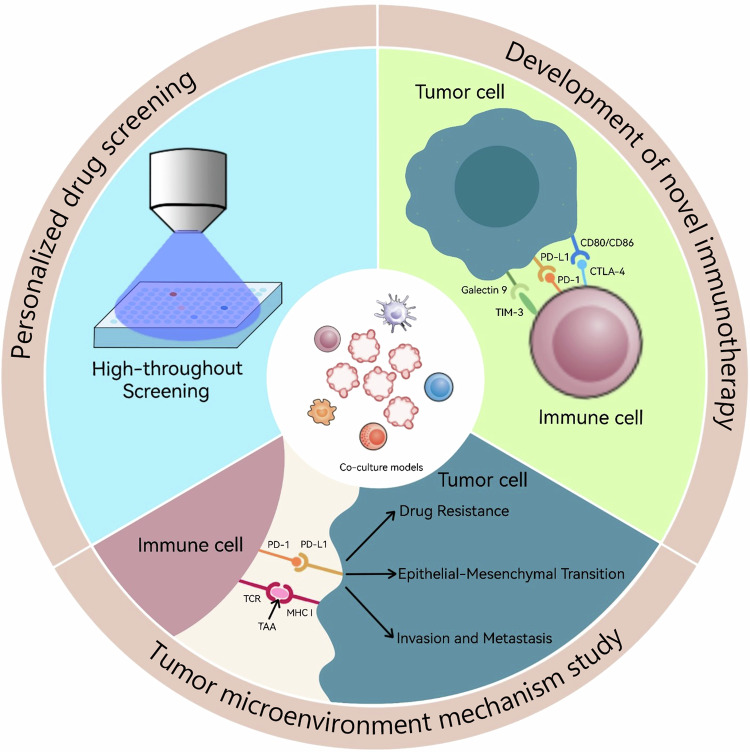
Application and prospects of co-culture of tumor organoids and immune cells. The diagram illustrates the applications of co-culturing tumor organoids with immune cells, consisting of simulating the interaction between tumor cells and immune cells, studying tumor microenvironment mechanisms (such as immunity and drug resistance), screening personalized drugs (for instance, high-throughput screening), and developing novel immunotherapy (such as the development of immune checkpoint inhibitors).

**Fig. 3:**
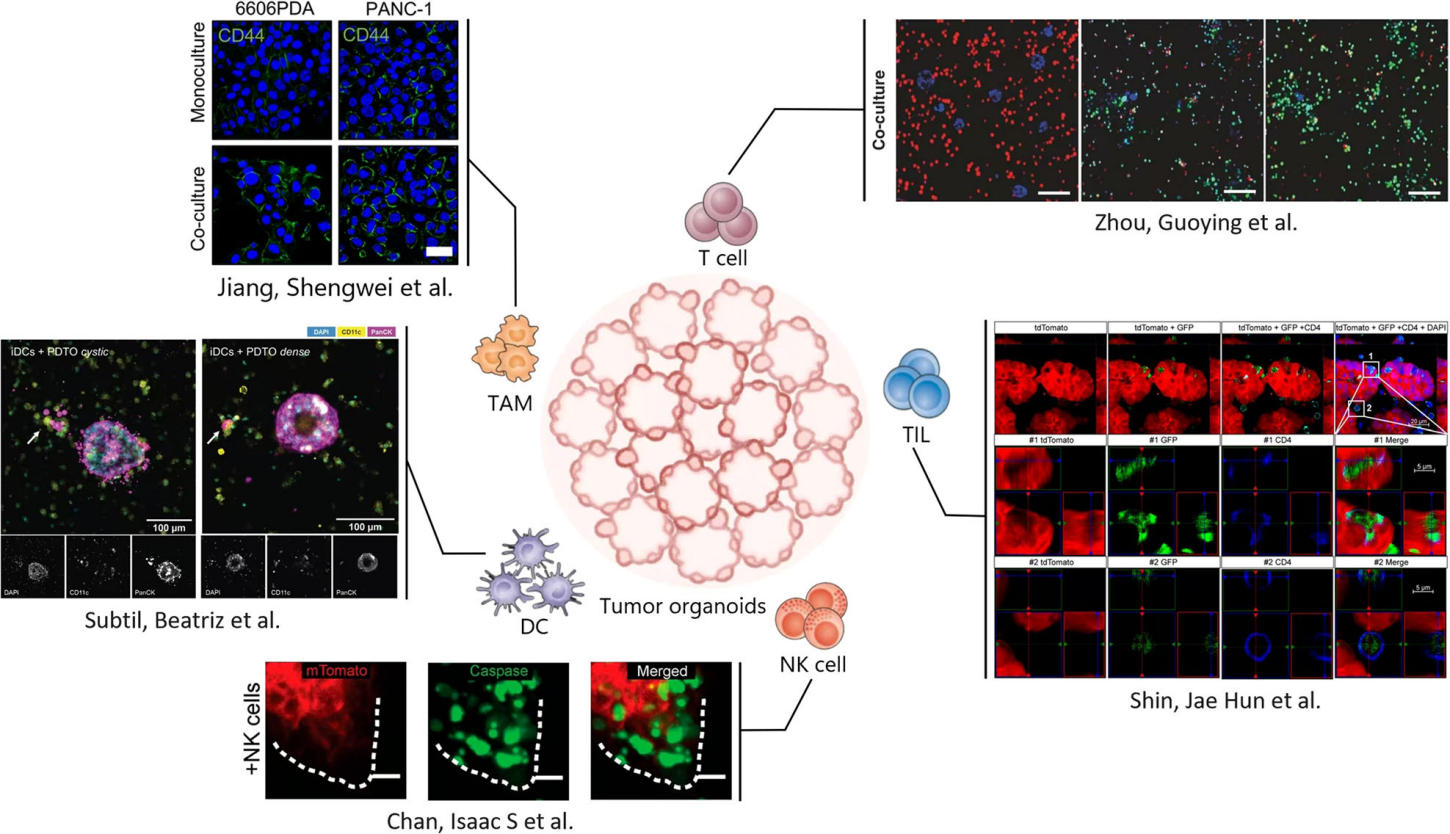
Future co-culture models. The figure depicts a possible future where in vitro co-culture more closely mimics the in vivo tumor microenvironment. Co-culture of organoids and immune cells can avoid the limitations of tumor organoid models and explore how cell-to-cell crosstalk affects immune responses. The top left figure is adapted from ref. [77], BioMed Central Limited. The top right figure is adapted from ref. [38], Nature Publishing Group Limited. The bottom right figure is adapted from ref. [61], National Academy of Sciences Limited. The bottom figure is adapted from ref. [68], Rockefeller University Press Limited. The middle left figure is adapted from ref. [81], Frontiers Media S.A. Limited. The surrounding experimental figures do not represent the actual situation of this model.

In tumor research, co-cultivation has emerged as a highly effective method for cultivating multiple cell types within a controlled environment (Fig. 1). Given that tumor organoids lack mature microenvironments, co-culture models that integrate tumor organoids with other cell types present in the tumor microenvironment can effectively replicate the complex interactions between cells within tumors [34]. By providing a more sophisticated platform to study tumor organoids in an environment that closely mimics actual tumor conditions, co-culture systems enable a more accurate simulation of intricate cellular interactions and signaling pathways [35] (Figs. 2 and 3). Consequently, co-culture has become an indispensable tool in tumor research, offering critical insights into the complexity of tumor behavior (Table 1). The establishment of co-culture models requires careful consideration of interactions between different cell types and the optimization of culture conditions. The composition and ingredients of the culture medium, cell density, culture time and other factors will affect the outcomes of co-culture model. In addition, the co-culture models can be utilized to simulate the effects of tumor treatments and interventions by incorporating specific factors, drugs, or gene-editing techniques. Common cell types used in tumor organoid co-culture models include tumor-associated fibroblasts, immune cells, endothelial cells, and neural cells. For instance, co-culturing tumor organoids with tumor-associated fibroblasts allows researchers to mimic the activation state of fibroblasts within tumors and investigate their roles in tumor development. Similarly, co-culturing tumor organoids with immune cells provides a platform to study tumor recognition and immune evasion mechanisms, as well as the immune system’s ability to monitor and attack tumors [20].

### T lymphocyte

T lymphocytes, as essential components of the immune system, play a critical role in combating tumors due to their ability to recognize and eliminate tumor cells. T cells are broadly categorized into two major subsets: CD8^+^ cells and CD4^+^ cells. CD8^+^ cells also referred to as cytotoxic T cells, are capable of directly identifying and destroying tumor cells. In contrast, CD4^+^ cells primarily function to regulate and coordinate immune responses, facilitating the activation and proliferation of CD8^+^ cells [36]. T cells are central to modern cancer immunotherapy, and therapeutic strategies targeting T cells have emerged as powerful tools in the fight against cancer. Groundbreaking scientific discoveries in the molecular and cellular biology of T cells have laid the foundation for innovative cancer treatment approaches, including immune checkpoint blockade, adoptive cell therapy, and cancer vaccine development [37]. The co-culture model of tumor organoids and T cells holds significant promise for advancing our understanding of tumor immune responses and for predicting the efficacy of immunotherapies.

Significant advancements have been made in the field of co-culturing tumor organoids with T cells, which are worth highlighting. Various tumor organoid models and co-culture systems incorporating T cells have been developed and reported in recent studies. For instance, Zhou et al. co-cultured patient-derived cholangiocarcinoma organoids with pre-activated peripheral blood mononuclear cells (PBMCs), establishing an immune cytotoxicity model specific to cholangiocarcinoma [38]. Similarly, Tsai et al. generated pancreatic cancer-responsive T cells by co-culturing peripheral blood lymphocytes with pancreatic cancer organoids. This work demonstrated the utility of co-culture technology in studying stromal and immune interactions within the tumor microenvironment, particularly in the context of pancreatic cancer [6]. Holokai et al. further advanced this approach by co-culturing mouse and human-derived autologous organoids with immune cells to create a preclinical model for pancreatic ductal adenocarcinoma. This model was used to evaluate the efficacy of immunotherapy and evaluated the consistency of these results with clinical outcomes [39]. Furthermore, Wang X and colleagues leveraged microsatellite stable CRC humanized mouse models and co-culture systems of colorectal cancer organoids with CD8^+^ T cells to demonstrate that *Fusobacterium nucleatum*, a CRC-associated pathogen, enhances the sensitivity of microsatellite stable CRC to immunotherapy, offering novel directions for clinical treatment [40]. To broaden the scope of tumor types studied in co-culture models with T cells and to validate the response of diverse tumors to immunotherapy, Chakrabarti et al. co-cultured mouse-derived gastric cancer organoids with autologous immune cells, studying the role of the PD-L1/PD-1 axis in anti-tumor immunotherapy [41]. Kükköse et al. developed an innovative model for spontaneous multiorgan metastasis in high microsatellite instability CRC tumors by transplanting patient-derived organoids into the cecum of humanized mice and analyzed the relationship between the therapeutic effects of anti-PD-1 and anti-CTLA-4 and the immune background of primary tumor, liver metastasis and peritoneal metastasis [42]. Moreover, co-culture models involving T cells have been reported for CRC, cervical squamous cell carcinoma, and non-small cell lung cancer, where fresh tumor biopsy samples were cultured to establish and expand tumor organoids [5, 43, 44].

The study of tumor organoids co-cultured with T cells provides a unique platform to study the immune response of tumors and predict the effect of immunotherapy [45, 46], which further promotes the development and optimization of immunotherapy [47–49], and also paves the way for the development of personalized therapies [50]. However, the co-culture of organoids and T cells currently lacks standardization and suffers from limited reproducibility. To enhance the predictive accuracy and clinical relevance of these models, future efforts must focus on more accurately simulating the tumor microenvironment and conducting cancer research at the individual level. Addressing these challenges will be critical for improving the utility and reliability of this promising methodology.

### CAR T cell therapy

Chimeric antigen receptor (CAR) T cell therapy represents a groundbreaking advancement in cancer treatment, leveraging genetic engineering to modify a patient’s T cells, thereby enabling them to identify and destroy cancer cells. At the core of this therapy are chimeric antigen receptors, which are engineered receptor proteins that confer new abilities to T cells, allowing them to target and recognize tumor-specific antigens. CAR T cell therapy has shown significant efficacy in certain types of cancer treatment, particularly hematologic malignancies such as acute lymphoblastic leukemia and specific forms of non-Hodgkin lymphoma. However, its effectiveness in solid tumors remains less clear, primarily due to challenges such as the infiltration, persistence, penetration, and inhibitory activity of CAR T cells within the tumor microenvironment [28]. Emerging research indicates that combining CAR T cell therapy with immune checkpoint inhibitors may hold promise for addressing solid tumors. Besides, the use of organoid platforms offers a potential avenue for replicating the complex tumor microenvironment.

Researchers have explored the co-culture of neuroblastoma organoids with CAR T cells to simulate the infiltration of CAR T cells into solid tumors, such as glioblastoma. By optimizing the ratio of CAR T cells to tumor cells, they successfully simulated and observed the infiltration, proliferation, and cytokine release during T cell activation. Moreover, they evaluated T cell invasion and proliferation, as well as the potential loss of target antigens in tumor cells and their subsequent killing by CAR T cells [51]. This study suggests that the co-culturing of tumor organoids with CAR T cells could serve as a preclinical model to assess CAR T cell efficacy and predict treatment response. Following this, Chen et al. successfully established an in vitro preclinical testing platform using co-culture models to evaluate CAR T cell-mediated cytotoxicity against mucin1-targeting bladder cancer organoids [52]. Numerous studies have demonstrated the potential of CAR T cells in tumor therapy, demonstrating their transition from laboratory research to clinical applications through co-culture models of tumor organoids and CAR T cells [53–55]. These models also enable the evaluation of treatment responses for solid tumors with specific targets. For instance, one study established a co-culture system using autologous HBV-positive hepatocellular carcinoma organoids with CD39^+^ HBV-CAR T cells or CD39^+^ personalized tumor-reactive CD8^+^ T cells to assess their anticancer efficacy [56]. In another study, Martins P et al. identified high expression of the Ephrin receptor A3 in glioblastoma and designed an Ephrin receptor A3-targeted CAR to evaluate its therapeutic potential against patient-derived glioblastoma organoids [57]. Furthermore, CRC organoids have been co-cultured with EGFRvIII-CAR NK-92 cells to create a platform for identifying target antigens and assessing the anti-cancer activity of CAR-NK-92 cells [14].

These studies offer the significant advantage of comprehensively capturing the molecular and cellular mechanisms underlying immunotherapy. However, challenges such as poor infiltration and persistence of CAR T cells, tumor heterogeneity, and genetic instability result in substantial variability in the co-culture of CAR-derived cells and tumor organoids when predicting efficacy and cytotoxicity. Notably, only a limited number of clinical trials have hitherto assessed the anticancer effects of CAR-engineered lymphocytes on cancer organoids, such as the anticancer effect of CAR macrophages in organoids derived from breast cancer patients (NCT05007379). Research in this area remains in its early stages, necessitating further exploration to refine this model and broaden its applications in cancer research.

### Tumor-infiltrating lymphocytes

Tumor-infiltrating lymphocytes (TILs) are naturally occurring lymphocytes that can penetrate the tumor microenvironment [58]. In human malignant tumors, the presence of TILs is common and serves as a marker of the host immune system’s response to tumor cells, reflecting the dynamic process of “cancer immune editing” [59]. As an effective immune defense mechanism against various malignancies, TILs are considered superior to other immune cells due to their capacity to generate a large number of T-cell receptor (TCR) clones to recognize a wide range of heterogeneous tumor antigens, offering superior therapeutic effects over TCR-T cells and CAR-T therapy [60]. Alternatively, the development of TILs as therapeutic agents faces various obstacles, including the inhibitory effects of the immune microenvironment and antigen mutations. In addition, the tumor microenvironment contributes significantly to promoting immune suppression. These factors collectively complicate the utilization of TILs as an effective therapeutic approach.

Shin JH et al. developed a novel model in which colorectal cancer organoids were co-cultured with TILs. Their findings revealed that lymphocyte membrane proteins are transferred to cancer cells via phagocytosis, a process that is time-dependent and relies on actin polymerization and PI-3 kinase signaling [61]. Lingling Ou et al. constructed patient-derived melanoma organoids and found that TILs significantly expanded when co-activated with IL-2 and anti-PD-1 antibodies, and could better infiltrate the melanoma organoid model. Furthermore, the study confirmed that the small molecule Navitoclax could enhance the immunotherapeutic effects of TILs, leading to the death of cancer cells within the model [62]. Hua Huang et al. established a biobank of patient-derived organoids comprising 67 heterogeneous cervical cancer cases and selected seven organoid samples for co-culture with TILs and specific killing experiments. The study successfully evaluated the responses of different organoid samples to TILs, revealing their potential value in personalized treatment strategies [63]. Zhilang Li et al. developed a co-culture platform designed to enrich and expand tumor-specific TILs in vitro by repeated stimulation with autologous tumor organoid cells derived from CRC patients. Their work demonstrated that organoid-autologous TIL co-culture serves as an effective system for identifying tumor-specific TCRs for TCR-T therapy [64].

The research highlights how cancer cells acquire immunomodulatory molecules through a co-culture system. However, the use of TILs for tumor treatment is still limited. More research is needed to address the clinical challenges of using TILs. Using tumor organoid-immune co-culture models for TIL research allows for a more accurate assessment of the role of TILs in tumor therapy. Moreover, this co-culture system provides valuable insights into the therapeutic value of TILs, their underlying mechanisms, and optimal application strategies.

### Natural killer cell

Natural killer cells are an essential component of the immune system, playing a critical role in identifying and eliminating tumor cells and virus-infected cells within the body. NK cells play a crucial role in tumor immunology, as they can not only directly eliminate cancer cells but also regulate anti-tumor immunity by producing chemokines and dendritic cells, recruiting and presenting tumor neoantigens to CD8^+^ T cells in lymph nodes. Furthermore, NK cells possess the ability to interact with T cells [65], either directly or indirectly enhancing or, in some cases, impairing T cell responses. The synergistic relationship between T cells and NK cells can directly result in the elimination of cancer cells [66]. As research advances, the importance of NK cells in tumor immunotherapy is becoming increasingly evident. By co-culturing tumor organoids with NK cells, researchers are able to investigate the mechanisms by which NK cells recognize and eliminate tumor cells, as well as how tumor cells develop strategies to evade NK cell-mediated attacks. This experimental model helps us better understand the immune evasion mechanisms of tumors and design more effective immunotherapy strategies.

In their study, Isaac S. Chan and Andrew J. Ewald described a co-culture method involving NK cells and tumor organoids to capture the interactions between these two cell populations. Their methodology involved isolating NK cells from tumors or human PBMCs and using positive selection to ensure the purity of the NK cell population. A 3D culture system was employed to examine how NK cells recognize and respond to specific characteristics of metastatic cancer cells. Furthermore, this co-culture platform was shown to be effective for screening the efficacy of drug inhibitors [67]. A separate study conducted at Johns Hopkins University investigated the interaction between breast cancer cells and NK cells. In this study, NK cells freshly isolated from healthy mice were activated using IL-2 or IL-15 and subsequently co-cultured with breast cancer-derived organoids in a 3D collagen I gel. The findings revealed that healthy NK cells significantly reduced the invasion, growth, and colony formation of tumor organoids in both the mouse mammary tumor virus-the Polyoma Virus middle T antigen and C3(1)-Tag mouse breast tumor models. Notably, healthy NK cells preferentially targeted keratin 14-positive cancer cells through direct cell-mediated cytotoxicity [68]. Another study co-cultured NK cells with pancreatic ductal adenocarcinoma (PDAC) organoids to investigate the impact of PDAC tumor cells on the phenotype and function of NK cells. Peripheral NK cells isolated from PDAC patients exhibited diminished cytotoxic activity and low levels of IFN-γ expression while displaying elevated levels of intracellular IL-10. This phenotypic profile was further confirmed in primary NK cells following co-culture with PDAC organoids [69].

These studies have investigated the interaction mechanisms between NK cells and tumor organoids; however, a notable limitation is that they remain in the early stages and lack sufficient depth. In addition, current research primarily focuses on exploring biological mechanisms through co-culture models. We anticipate that future studies will leverage such models to test novel immunotherapy drugs and further investigate immune escape mechanisms in tumors.

### Macrophage

Macrophages are among the most abundant cell types in the tumor microenvironment and are known for their ability to phagocytose and eliminate pathogens [70]. Tumor-associated macrophages (TAMs) play a key role in the tumor microenvironment and have significant impacts on tumor growth, metastasis, and treatment responses [71]. Nevertheless, the specific roles of TAMs in tumor progression and their interactions with tumor cells remain incompletely understood. TAMs exhibit a wide range of dynamic functions within the tumor microenvironment, displaying both pro-tumorigenic and anti-tumorigenic properties depending on their polarization and activation states [72]. Strategies targeting TAMs in cancer treatment include depleting TAMs, reprogramming them towards an anti-tumor phenotype, or inhibiting their recruitment to tumor sites. These approaches can be combined with other treatment modalities such as chemotherapy, radiotherapy, anti-angiogenic agents, and immunotherapy [73]. Nonetheless, translating TAM-targeted therapies into effective treatments remains a challenge. Researchers have increasingly turned to 3D co-culture systems to simulate the tumor microenvironment and study the behavior of TAMs.

Linde et al. co-cultured macrophages with human skin squamous cell carcinoma organoids and found that tumor cells and the extracellular microenvironment, including stromal cells and extracellular matrix, influenced the changes in macrophage phenotype [74]. In a separate study, researchers developed a multi-layer microfluidic chip designed to co-culture patient-derived hepatocellular carcinoma organoids with macrophages for the prediction of responses to immunotherapy drugs in an in vitro setting [75]. Both studies demonstrated that the use of a co-culture model could better simulate the in vivo conditions, but the interaction mechanism between tumor organoids and macrophages has not been described. Another study co-cultured CRC organoids with macrophages to simulate in vivo interactions between tumor cells and immune cells within the tumor microenvironment. The researchers discovered that high expression of NAD-dependent deacetylase sirtuin-1 in CRC cells promoted macrophage migration and invasion. Macrophages derived from sirtuin-1-high CRC exhibited an M2 phenotype, which is typically associated with immunosuppression. Co-culture experiments showed that the expression of sirtuin-1 in CRC could modulate the interactions between TAMs and CD8^+^ T cells, ultimately leading to a suppressed anti-tumor immune response [76]. Jiang et al. not only used the macrophage-organoid co-culture model to elucidate the involvement of the macrophage-CCL5-Sp1-AREG loop in the interplay between macrophages and pancreatic cancer cells, but also verified that Gefitinib, Verteporfin, and Maraviroc, especially inhibitors such as Mithramycin, showed promising results in the co-culture model, indicating their potential to treat pancreatic adenocarcinoma and verified in mouse models [77]. This study effectively utilized a co-culture model to explore how sirtuin-1 overexpression promotes macrophage migration and invasion. However, a notable limitation is the lack of in vivo validation, which would be essential to confirm these findings in a more physiologically relevant context.

TAMs represent a highly diverse and adaptable cell population, and our understanding of their exact roles across various tumor types and stages remains incomplete. Moving forward, it is anticipated that the use of 3D co-culture models will provide deeper insights into the interactions between tumor cells and macrophages. Such models may help elucidate how these interactions influence tumor progression and treatment responses, thereby offering promising avenues for the development of novel therapeutic strategies targeting TAMs.

### Dendritic cell

Dendritic cells (DCs) are highly specialized antigen-presenting cells that can capture and process antigens, and then present these antigens to T cells to activate immune responses. Beyond their antigen-presenting function, DCs are also pivotal in regulating immune activity. Through the secretion of cytokines, they can either activate or suppress T cell responses, thereby shaping the intensity and direction of immune responses [78]. In recent years, DCs have attracted significant interest in the field of cancer therapy. Researchers are exploring strategies to harness the potential of DCs to either enhance the immune system’s ability to target and destroy tumors or suppress immune responses that may contribute to tumor growth [79]. Co-culture tumor organoids with DCs can simulate the tumor microenvironment more realistically in vitro and allow real-time study of the interactions between DCs and tumor cells [80].

Subtil et al. conducted a study in which they co-cultured patient-derived metastatic CRC organoids with DCs to investigate their interactions. The results showed that metastatic CRC organoids modulate the behavior, phenotype, and function of monocyte-derived DCs within a collagen matrix. The 3D co-culture model demonstrated high cell viability and extensive interactions between DCs and tumor organoids [81]. The research team also evaluated the plasticity of tumor-induced cDC2 phenotypes. Following co-culture with CRC organoids, they observed that approximately 46% of cDC2 cells expressed CD14. These CD14 + cDC2 cells displayed impaired T cell proliferation and activation. The researchers suggested that this cDC2 phenotypic switch might contribute to the disruption of the immune system by CRC tumors, potentially influencing anti-tumor immunity and treatment efficacy [82]. A recent clinical trial on patients with glioblastoma co-cultured Dendritic cells—Cytokine Induced Killer cells (DC-CIK) with glioblastoma organoids. The trial confirmed an increase in the specific lysis of tumor cells after co-culture with DC-CIK cells. Mechanistic studies further revealed that the targeting mechanism of DC-CIK cells on glioblastoma cell models involved a significant increase in apoptosis and elevated secretion levels of IFN-γ, rather than TNF-α [83]. Yike Hou et al. employed a co-culture model of melanoma organoids with DCs to validate the efficacy of a nanovaccine developed by their research team. The nanovaccine was shown to effectively stimulate the maturation of bone marrow-derived DCs, thereby promoting the maturation and activation of T cells. This process ultimately led to the robust production of immunologically significant cytokines and effectively inhibited the growth of melanoma [84].

The co-culture of tumor organoids with DCs offers a model that more closely mimics physiological conditions. This approach facilitates the discovery of novel therapeutic strategies aimed at preventing tumor-induced immunosuppression or restoring the anti-tumor activity of immune cells. Further exploration of the interactions between DCs and tumor organoids may unveil key mediators and potential therapeutic targets in tumor progression.

## Co-culture methods of tumor organoids with immune cells

There are several methods for constructing tumor organoids co-cultured with immune cells (Fig. 1), which are selected based on the research objectives and the specific types of tumor organoids and immune cells involved [85]. Autologous co-cultures, where tumor organoids and immune cells are derived from the same individual, most closely mimic the in vivo environment. However, the establishment of autologous co-cultures is often limited due to tissue availability. Therefore, most studies primarily utilize allogeneic systems, in which immune cells are obtained from donors different from those of the tumor organoids. It is important to note that allogeneic systems can trigger strong immune reactions due to HLA mismatches, which might obscure specific responses against tumor-associated antigens [86].

Regardless of whether the tumor organoids and immune cells in the co-culture system are autologous or allogeneic, the most extensively studied approach is direct co-culture (Fig. 1). In this method, tumor organoids and immune cells are placed in direct physical contact, facilitating direct interaction and communication between the two cell types. However, due to the differing culture requirements for tumor organoids and immune cells, it is often necessary to conduct preliminary experiments to optimize the conditions that support the growth and viability of both cell types. Generally, tumor organoids and immune cells are first cultured separately in their respective specialized media before initiating co-culture. While this approach is relatively straightforward and easy to implement, it presents challenges in precisely controlling cellular interactions, which may introduce variability and potentially impact the experimental outcomes.

Another commonly used method for co-culturing tumor organoids with immune cells is the Transwell co-culture system (Fig. 1). The Transwell technique is a well-established experimental approach that enables two or more cell types to be cultured within the same system while being physically separated by a semi-permeable membrane. This membrane allows small molecules, cytokines, and other soluble factors to pass through but prevents direct cell-to-cell contact. As a result, this method is suitable for studying indirect cellular communication and interactions, rather than those requiring direct cell-cell contact. However, the presence of the membrane may influence intercellular signaling pathways, potentially limiting the accuracy of simulating in vivo immune responses. In a typical setup, tumor organoids are cultured at the bottom of the dish, while immune cells are placed on a Transwell insert equipped with a semi-permeable membrane, positioned above the organoids. The co-culture is maintained under appropriate media and conditions to support the growth and interaction of both cell types [87].

In recent years, co-culture techniques utilizing microfluidic chip technology have also received widespread attention (Fig. 1). Microfluidic devices enable researchers to precisely regulate cell interactions and the delivery of soluble factors [88], offering a powerful tool for studying dynamic interactions between immune cells and tumor organoids. On these chips, tumor organoids and immune cells can be manipulated at the micrometer scale, allowing for the simulation and studying complex interactions between tumors and the immune system. Microfluidic-based co-culture systems provide a physiologically relevant model, facilitating the study of complex tumor-immune interactions within a microenvironment that mimics cell-to-cell communication. However, this approach has its drawbacks, including the need for more sophisticated technical expertise, higher costs, and specialized equipment and skills. Existing research is mainly based on these three methods (Table 1). Each co-culture method has its own strengths and limitations, and researchers should carefully select the most appropriate method based on their research goals and experimental conditions.

## Application of tumor organoid-immune co-culture models

Tumor organoid-immune co-culture models have important applications in several fields and provide an essential experimental platform for studying the immune evasion mechanisms of tumors, the mechanisms of immunotherapy, and the development of new immunotherapeutic strategies (Fig. 2). These models enable researchers to study how tumor cells evade the immune system by observing immune cell behavior within tumor organoids, such as alterations in antigen expression and the secretion of immune-suppressive factors. Ko et al. established 32 genetically engineered esophageal organoids and utilized single-cell RNA sequencing to analyze the transcriptome phenotypes of these organoids and the chemokines they released. Their work identified that the deletion of TP53, CDKN2A, and NOTCH1 serves as a key genetic determinant in inducing esophageal tumors and immune escape [89]. These findings enhance our understanding of tumor immune evasion mechanisms and provide a theoretical foundation for the development of new immunotherapeutic approaches. Moreover, tumor organoid-immune cell co-culture models are valuable for screening and evaluating new immunotherapeutic drugs. For instance, researchers can assess the efficacy of these drugs by observing whether they effectively activate immune cells and induce tumor cell death within the co-culture system. For example, Norkin et al. employed CRC organoids to evaluate the efficacy of several drug classes not previously reported for CRC treatment, including antipsychotic phenothiazines, cholesterol-lowering statins, antimycotic conazoles, selective estrogen receptor modulators, and antihistamines. Their study identified ifenprodil, opipramol, perphenazine, and toremifene as promising candidates with a favorable therapeutic window for targeting human CRC, warranting further in vivo validation [90]. Such findings provide significant preclinical data to support future clinical trials of these drugs. Besides, tumor organoid-immune cell co-culture models can be applied to personalized immunotherapy. For example, researchers can isolate tumor cells from a patient’s tissue, construct patient-specific tumor organoids, and co-culture them with the patient’s own immune cells to evaluate the immune system’s response to the tumor and the effectiveness of various immunotherapeutic strategies. Van de Wetering et al. constructed patient-derived CRC organoids to test the activity of cetuximab in KRAS wild-type organoids. Their results, consistent with clinical observations, highlighted the potential of tumor organoids to directly assess the drug sensitivity of tumors in a personalized treatment approach [91].

These research findings hold significant potential for informing personalized immunotherapy strategies for patients. The innovative approach of co-culturing tumor organoids with immune cells offers a powerful tool to simulate the tumor microenvironment and investigate the complex interactions between tumors and the immune system. The applications of this research method not only help us gain a deeper understanding of the immune evasion mechanisms of tumors but also provide a vital experimental basis for the development of new immunotherapeutic strategies and personalized immunotherapy plans.

## Challenges, opportunities and prospects

The co-culture of tumor organoids with immune cells offers a valuable model for simulating the tumor microenvironment, serving as a critical tool for investigating the interactions between tumors and the immune system. However, the current reliance on components such as purified growth factors, conditioned media, and animal-derived sera for cancer organoid culture media presents significant challenges. These components are often costly, lack reproducibility, and exhibit high heterogeneity, which hinders the ability to achieve accurate patient-specific modeling of the tumor microenvironment [92]. The most widely used matrix for tumor organoid culture is the Engelbreth-Holm-Swarm (EHS) matrix. While the EHS matrix supports the 3D culture of human tumor organoids, it is derived from animal sources, leading to substantial batch-to-batch variability. Moreover, it contains poorly defined xenobiotic impurities that can unpredictably influence organoid phenotypes [93]. For example, Matrigel, a commonly used EHS matrix, comprises over 14,000 unique peptides and nearly 2000 different proteins, many of which are known to alter tumor cell behavior. Even with growth-factor-reduced formulations of the EHS matrix, the similarity in protein content between batches remains limited, at approximately 53% [94, 95]. In recent years, collagen type I matrices have emerged as a more affordable and biomimetic alternative to the EHS matrix for in vitro tumor organoid models. However, since collagen is also derived from animal sources, it shares similar limitations, including batch-to-batch variability and the presence of undefined xenobiotic impurities [92]. Chemically synthesized hydrogels can provide a more precise growth environment for tumor organoids by regulating their composition and properties. Compared to animal-derived hydrogels, their components are easier to control, which can avoid batch-to-batch variations. In the context of drug screening and the development of novel therapies, chemically synthesized hydrogels can better simulate the in vivo environment, offering more accurate drug response information [96, 97].

The development of tumor organoids and the isolation and expansion of immune cells require sophisticated experimental techniques and strict experimental conditions, which place high demands on both the technical expertise and equipment capabilities of the research facility. Time, standardization, and reproducibility are three critical factors that must be carefully considered when working with any in vitro tumor model [98]. In addition, the co-culturing process of tumor organoids with immune cells necessitates the simulation of various elements within the tumor microenvironment, including intercellular interactions, cell-matrix interactions, oxygen levels, and nutrient supply. These requirements add considerable complexity to the experimental setup. While the co-culturing model of tumor organoids with immune cells can mimic certain aspects of the tumor microenvironment, it remains challenging to fully replicate the intricate conditions found within the human body. For instance, the tumor microenvironment includes other cell types, such as vascular cells and fibroblasts, as well as a variety of cytokines and chemical signals, which are often overlooked in current co-culturing models [85, 98, 99]. To more accurately simulate the tumor microenvironment, it is essential to construct the physical architecture of both components in the co-culture system, incorporate a broader range of immune cell types, and select a culture medium that supports the growth of diverse cell populations. Besides, the results obtained from co-culturing models should be validated through in vivo experiments to ensure their relevance and reliability [81]. Organ-on-a-chip technology can provide a more dynamic microenvironment, such as vascular perfusion and fluid dynamics, which is closer to the conditions inside the human body [100, 101]. Similarly, 3D bioprinting technology can precisely replicate the physical structure of tumor tissues, from macroscopic to microscopic structures, improving the biological authenticity of the model [102]. By combining these technologies with tumor organoid-immune cell co-culture models, we can better understand the complexity of the tumor microenvironment and improve our understanding and treatment of tumors.

Despite these challenges, the co-culturing of tumor organoids with immune cells as a research method presents numerous opportunities. Scientists aim to develop more sophisticated and realistic co-culture models by incorporating a wider variety of cell types and more complex cytokine networks to better simulate the tumor microenvironment (Fig. 3). Furthermore, the integration of big data and artificial intelligence technologies with these organoid-immune co-culture models holds significant promise. This synergy could enhance our understanding in several ways: by aggregating data from diverse sources to provide a comprehensive view of the intricate interactions within the tumor microenvironment; by employing machine learning algorithms to identify critical factors and patterns related to tumor growth, development and immune response ; by predicting the efficacy of various treatment strategies for specific tumor types and assessing the risk of disease recurrence, thereby aiding clinicians in crafting more effective treatment plans; and by refining experimental conditions through virtual modeling to improve the stability and reproducibility of co-culture models [43, 103]. Real-time monitoring and data analysis enable researchers to promptly adjust experimental parameters, ensuring the precision of the outcomes. Besides, big data and artificial intelligence can foster interdisciplinary collaboration by establishing standardized platforms for data sharing, which would consolidate information from different laboratories and clinical trials, enhancing the model’s accuracy [104]. However, in the research of tumor organoids with big data and artificial intelligence, it is necessary to follow the ethical review system to ensure the legitimacy and ethical integrity of the research [105]. With the progress of tumor immunology, we have gained a deeper understanding of the interaction between tumors and the immune system. A recent study found that CXCR3 expression in regulatory T cells plays a crucial role in tumor immunity. This expression enables regulatory T cells to interact with DCs in tumors, thereby affecting the response of CD8^+^ T cells to tumors. This finding provides a new research direction for cancer immunotherapy [106]. Another study transplanted human intestinal organoids under the renal capsule of mice with humanized immune systems, creating intestinal organoids containing immune cells and demonstrating crosstalk between intestinal epithelial cells and immune cells [107]. By simulating the co-culture of tumor organoids with immune cells, we can better understand how this interaction affects immune responses and potentially bring more effective treatments for cancer patients.

## Synthesis

Research on co-culture models of tumor organoids with immune cells offers a powerful tool for understanding the interaction between tumors and the immune system, as well as for developing and optimizing immunotherapy strategies. By employing this approach, we can replicate the tumor microenvironment in vitro, allowing us to observe how immune cells recognize and target tumor cells, as well as how tumor cells develop mechanisms to evade immune detection. These insights are invaluable for designing more effective immunotherapies and predicting patient responses to such treatments. Nonetheless, co-culture models of tumor organoids with immune cells are not without limitations. Establishing and maintaining these models demand advanced experimental techniques and stringent conditions. Besides, these models cannot fully replicate the complexity of the in vivo environment. Despite these challenges, co-culture systems remain a highly valuable research tool. As scientific and technological methods continue to evolve, we anticipate the development of more complex and physiologically relevant models that better mimic the tumor microenvironment. In addition, with the development of big data and artificial intelligence technologies, data analysis and model predictions will enable more accurate evaluations of immunotherapy efficacy and predictions of patient responses to immunotherapy.

## Conclusions

This article presents a comprehensive review of current research on co-culture models involving tumor organoids and immune cells. Such co-culture models serve as invaluable tools for enhancing our understanding of the complex interactions between tumors and the immune system. Furthermore, they provide critical insights that can guide the development and optimization of immunotherapy strategies. While this research approach is not without its challenges, we anticipate that ongoing technological advancements will help address these limitations. To harness the potential of this model, it is imperative to pursue further in-depth studies, which will ultimately advance the field of immuno-oncology and its clinical applications.
